# Can Pacing Be Regulated by Post-Activation Potentiation? Insights from a Self-Paced 30 km Trial in Half-Marathon Runners

**DOI:** 10.1371/journal.pone.0150679

**Published:** 2016-03-02

**Authors:** Sebastián Del Rosso, Edilberto Barros, Laís Tonello, Iransé Oliveira-Silva, David G. Behm, Carl Foster, Daniel A. Boullosa

**Affiliations:** 1 Grupo de Investigación en Estilos de Vida y Estrés Oxidativo, CenIHN - Escuela de Nutrición, DAAN, Hospital Nacional de Clínicas, Facultad de Cs. Médicas, Universidad Nacional de Córdoba, Córdoba, Argentina; 2 Pós-Graduação Stricto Sensu em Educação Física, Universidade Católica de Brasília, Águas Claras, Brazil; 3 School of Human Kinetics and Recreation, Memorial University of Newfoundland, St. John's, Newfoundland, Canada; 4 Department of Exercise and Sport Science, University of Wisconsin, La Crosse, Wisconsin, United States of America; University of Rome, ITALY

## Abstract

**Purpose:**

Given the co-existence of post-activation potentiation (PAP) and fatigue within muscle, it is not known whether PAP could influence performance and pacing during distance running by moderating fatigue. The aim of this study was to assess the influence of PAP on pacing, jumping and other physiological measures during a self-paced 30 km trial.

**Methods:**

Eleven male endurance-trained runners (half-marathon runners) volunteered to participate in this study. Runners participated in a multi-stage 30 km trial. Before the trial started, determination of baseline blood lactate (bLa) and countermovement jump (CMJ) height was performed. The self-paced 30 km trial consisted of 6 × 5 km splits. At the end of each 5 km split (60 s break), data on time to complete the split, CMJ height, Rating of Perceived Exertion (RPE) and blood lactate were collected while heart rate was continuously monitored.

**Results:**

There was a significant decrease in speed (e.g. positive pacing strategy after the 4^th^ split, p<0.05) with a progressive increase in RPE throughout the trial. Compared with baseline, CMJ height was significantly (p<0.05) greater than baseline and was maintained until the end of the trial with an increase after the 5^th^ split, concomitant with a significant reduction in speed and an increase in RPE. Significant correlations were found between ΔCMJ and ΔSPEED (r = 0.77 to 0.87, p<0.05) at different time points as well as between RPE and speed (r = -0.61 to -0.82, p<0.05).

**Conclusion:**

Our results indicates that fatigue and potentiation co-exist during long lasting endurance events, and that the observed increase in jump performance towards the end of the trial could be reflecting a greater potentiation potentially perhaps counteracting the effects of fatigue and preventing further reductions in speed.

## Introduction

Performance in endurance events is the result of an athlete’s ability to achieve and maintain power output or speed over the course of a competition. This is achieved through strategies aimed at managing the rate of fatigue development and thus the reduction in speed which would be detrimental for performance [1, 2]. Consequently, ‘pacing strategy’ is the self-selected tactic used by athletes from the beginning of an event to distribute their work rate throughout the competition [1, 3, 4] in order to prevent premature fatigue and thus optimize performance [4, 5].

Pacing can be influenced by a number of factors, which can influence fatigue development and impair performance. In long-distance efforts (e.g. > 40 min) it has been suggested that pacing may be regulated by the perception of effort (often measured with Rating of Perceived Exertion [RPE] scales) where athletes adjust their running speed by comparing moment-to-moment the actual with the expected RPE for a given distance [6–8]. Several models have been proposed to explain fatigue in endurance sports [9]. Yet, it is known that the magnitude and etiology of fatigue would depend on the exercise under consideration and it has been recommended that the study of muscle fatigue should address both the perceived effort and the decline in force that occurs during sustained activity [10]. The main focus to explain how fatigue could influence pacing in endurance sports has been on centrally mediated mechanisms [3, 6, 11, 12]. However, the study of factors that integrate both peripheral and central fatigue with pacing strategies should also be of interest to better characterize endurance running events. This is important in light of evidence showing that group III/IV muscle afferents are involved in the development of both peripheral and central fatigue [13, 14]. Accordingly, it would be pertinent to consider that pacing could be regulated by mechanisms acting both at central and peripheral levels.

It has been pointed out that central fatigue alone cannot explain the entire muscle strength loss during and after prolonged running exercises [15] and that alterations at the neuromuscular level may also be involved. One potential factor relating fatigue and pacing could be the neuromuscular profile of the athletes. Since the classical work of Paavolainen and co-workers [16] it has been known that improving athlete’s muscle power through strength training improves endurance performance [17, 18]. More recently, Boullosa et al., [19] showed that, after a fatiguing running test, endurance-trained runners experienced an improvement in countermovement jump (CMJ) that correlated with an increase in peak power and a lower eccentric maximum strength loss during the CMJ. This finding was consistent with that reported by others [20] and it is believed to be associated with the co-existence of post-activation potentiation (PAP) and fatigue within the muscle. PAP is defined as an acute improvement of muscular performance characteristics as a result of the contractile history [21] and it has been suggested that the PAP response after running is specific for endurance-trained athletes [19, 22]. The most important mechanism that has been proposed in literature to explain PAP is phosphorylation of myosin regulatory light chains, which increases Ca^2+^ sensitivity of the myofilaments [23]. Alternatively, acute changes in muscle architecture and increased recruitment of higher order motor units have been also proposed as potential mechanisms [21]. It appears that PAP has its greatest effect during submaximal contractions in which motor units are firing at relatively low frequencies, such as the case of endurance exercise [24]. Furthermore, it could be argued that PAP might counteract the onset of fatigue [24] at the peripheral level. In addition, given that submaximal fatigue is defined as the balance between fatigue-induced impairments and neuromuscular strategies to sustain performance [25], it is possible that other mechanisms may play a role. Among these mechanisms, those acting to facilitate muscle performance are likely to be of importance. At a supraspinal level, neural potentiation could be one of those factors facilitating the maintenance of muscle performance during prolonged activities, as it has been suggested that ongoing contractions facilitate motoneuron excitation and contributes to improved force production [25]. However, while the acute effect of PAP in endurance athletes after different running exercises has been reported [26, 27], it is not known whether or not PAP would influence performance and pacing during endurance activities.

After a 30 km trial in endurance trained runners, Millet and co-workers [28] observed a ~23.5% reduction in maximal voluntary contraction of the knee extensors concomitant with a decrease in voluntary activation, which was attributed to central fatigue. Additionally, they found no change in low frequency fatigue and suggested that exercise may have potentiated the contraction torque and hidden the low frequency fatigue. As previously mentioned, the contractile history of a muscle contributes to both fatigue and potentiation [29] and it has been pointed out that potentiation can occur after both maximal and submaximal efforts in endurance trained athletes [19, 26, 27, 30]. Moreover, a comparison of endurance and power trained athletes showed that submaximal exercise can offset fatigue in endurance but not in power athletes [31]. This important difference could be partially attributed to the increase in the content of fast myosin light chains in slow-twitch fibers in endurance trained subjects, an adaptation that likely increases the capacity of myosin light chain phosphorylation. Accordingly, despite the ongoing increase in central fatigue and the reduced muscle activation through central mechanisms, the potentiation effect could be of importance for long distance runners to maintain or prevent further reductions in pacing.

Accordingly, the main purpose of this study was to assess the pacing adopted by a group of endurance-trained runners during a self-paced 30 km trial, along with perceived exertion, jump capacity, lactate, and HR responses to evaluate whether these responses, and more specifically the expected jump potentiation, might influence pacing.

## Materials and Methods

### Participants

Eleven male trained endurance athletes (half-marathon runners) volunteered to participate in this study (Table 1). For an expected effect size of 0.5 (moderate effect) and a minimum of 6 measurements for most of the variables, a sample of 8 participants would yield a statistical power of 0.95. All runners had at least five years of experience and were in the middle of their training macrocycle. The runners were training a mean of 8.6 ± 0.4 sessions and ~152.6 ± 3.9 km (133 to 172 km) per week with a volume per training session ranging from 8 to 25 km. Before engaging in any test, participants were informed about the risks and benefits of the study and provided written informed consent. The protocol of the study was approved by the Ethics Committee of Catholic University of Brasilia.

**Table 1:** Characteristics of the half-marathon runners.

	X ± SD
Age (years)	28.5 ± 4.2
Body Mass (kg)	64.35 ± 7.54
Height (m)	1.72 ± 0.08
BMI (kg·m^2^)	21.5 ± 1.4
%Fat	7.83 ± 10.71
MAS (km·h^−1^)	20.45 ± 1.63
T_UMTT_ (min)	28.79 ± 2.81
HRrest (bpm)	57 ± 9
HRmax (bpm)	185 ± 9

Values are means ± SD. BMI = body mass index, MAS = maximum aerobic speed, T_UMTT_ = time to complete the UMTT (*Université of Montreal Track Test*), HRrest = heart rate at rest, HRmax = maximum heart rate.

### Procedures

**Fig 1:**
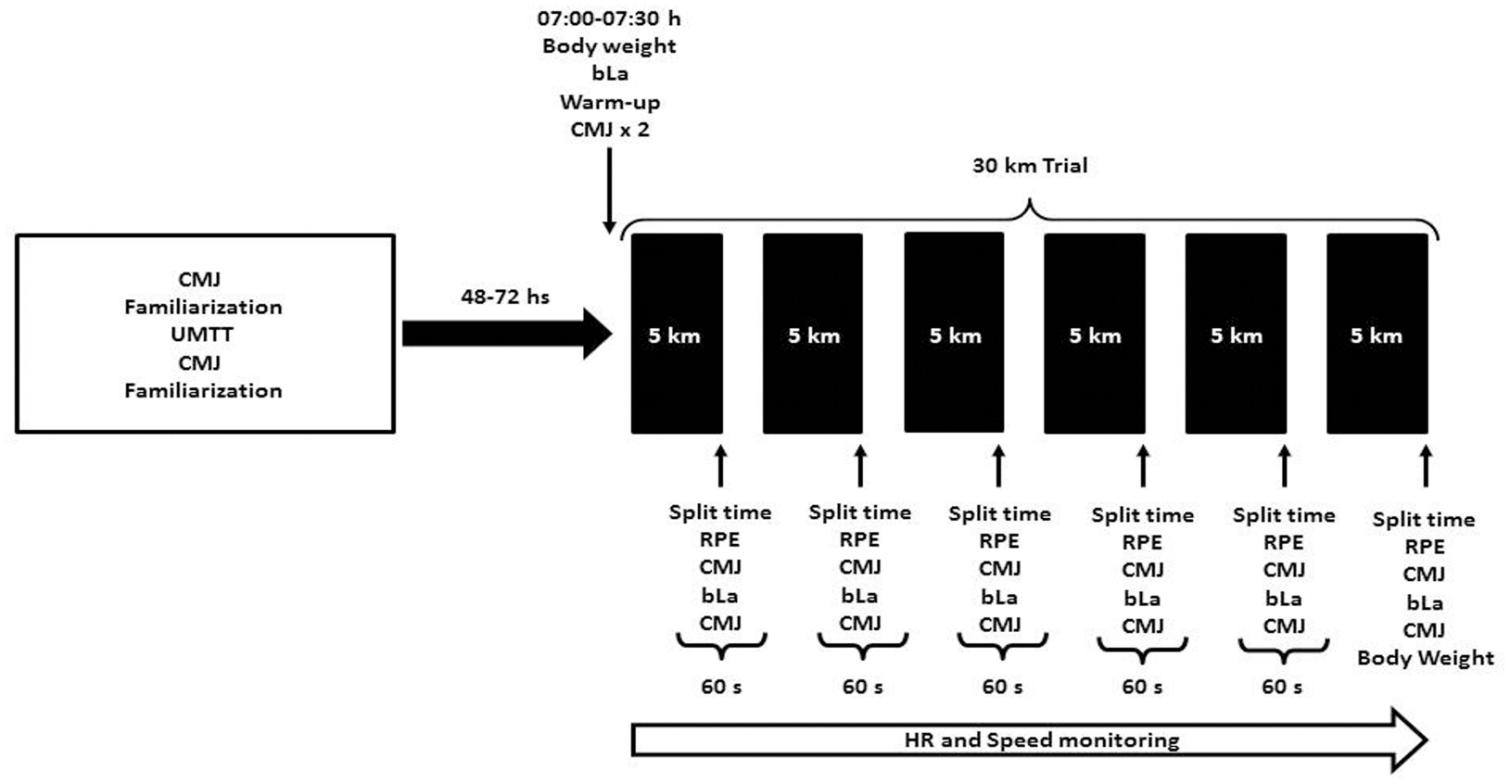
Study protocol. UMTT = Université of Montreal Track Test, CMJ = Countermovement Jump, RPE = rate of perceived exertion, bLA = blood lactate.

Runners were required to participate in two separate testing sessions. The first was devoted to anthropometric measurements [i.e., percentage of body fat (%BF)], the assessment of maximum aerobic speed (MAS) and familiarization with the CMJ protocol. During the second session, a self-paced 30 km trial was carried out which consisted of 6 × 5 km splits on a course within the University campus. This uncommon competitive distance was selected to avoid the possible influence of competitive experience on running performance. Based on a previous study [32], a multi-stage approach was applied for evaluating the evolution of neuromuscular performance through the whole trial. At the end of each 5 km split, data on CMJ height, RPE (6–20 scale [33]) and blood lactate (bLA) were collected. In the case of RPE, the participants were instructed to report the effort perception for the entire previous split (i.e., akin to session RPE). In addition, heart rate (HR) and speed were monitored. The sessions were separated by 48–72 h to avoid the effects of residual fatigue (Fig 1). The order of the start when various athletes were evaluated in the same day was based on the individual MAS previously measured (i.e., the fastest runner was the first to start the trial) and subsequent runners started the 30 km trial every 5 min to avoid the effect of having a “rabbit” on performance [34]. However, it is worth noting that although faster athletes never surpassed the slower ones, on some occasions they crossed each other along the course.

### Université de Montréal Track Test

The UMTT was carried out on a 400 m synthetic track and its protocol is described elsewhere [35] but the pacing was set using a bicycle equipped with a speedometer [26]. Briefly, the test started with a running speed of 8 km·h^−1^ and increased in 1 km·h^−1^every 2 min. Participants were instructed to run behind the bicycle and to avoid drafting. The test was terminated when the participants could no longer follow the pacing and distance ≈ 3 m from the bicycle. Maximum aerobic speed (MAS) was defined as the speed achieved in the last completed 2 min stage with recording of the total final time (T_UMTT_). Before and immediately after completing the UMTT the participants performed three CMJs separated by at least 15 s, in order to familiarize with the jump protocol.

### Countermovement Jump Height (CMJ)

CMJ was selected because is easy to perform and has been shown that it could be used to reflect the effect of PAP and fatigue in athletes [19]. CMJ was recorded using a contact platform connected to a digital timer through an interface (ChronoJump-BoscoSystem) [36, 37]. The flight time of each individual jump was recorded and converted automatically to jump height by the specific software that was connected to a personal computer [37]. In each CMJ, participants were encouraged to jump as high as possible with the arms in akimbo (both hands on the hips) position. The depth of each jump was freely chosen by the participants [19]. In addition, the athletes were instructed to maintain their knees extended during the landing phase.

### Self-Paced 30-km Trial

For the 30 km-trial, a 5 km course was demarcated inside the university campus. A global position system (GPS) monitor (Garmin Forerunner 305, Garmin Ltd., Olathe, KS, USA) with an accuracy of 0.05 m·s^−1^ in steady conditions was used by a researcher to measure the course to be completed by the runners. Participants arrived at the location at 07:00 h and were equipped with a heart rate (HR) monitor (Polar RS800 CX, Polar Electro Oy, Finland). Then, after resting quietly for 15 min, a blood sample was obtained for determination of baseline blood lactate (bLa) using a dry chemistry analyzer (Lactate Plus, Nova Medical, Waltham MA, USA). Body mass (BM) was measured using an electronic scale (Filizola ID 500, São Paulo, Brasil). Following these procedures, a standard warm-up was carried out consisting of 5 min of jogging at a personally selected intensity, dynamic stretches and two CMJs. Then, participants performed two maximal CMJ separated by 15 s to assess baseline CMJ height. The self-paced 30 km trial started at ~ 07:30 h and consisted of 6 × 5 km splits. The participants were instructed to run the entire course (i.e., 30 km) as fast as they can and were monitored by one researcher along the course. Each 5 km split started and finished at the same location. At the end of each split the runners stopped for measurements of bLA, RPE (6–20 scale [33]), and CMJ height (Fig 1). The order of the measurements was selected to minimize the time required to collect the data and was the following: split time, 1^st^ CMJ, bLA and RPE, 2^nd^ CMJ. The mean total time required for data collection at the end of each split was 60 s. In addition, HR was monitored continuously during the 30 km self-paced trial. The mean temperature and relative humidity during data collection was ~24.1°C and ~37.1%. During the trial, carbohydrate-electrolyte beverages (Gatorade^®^) were provided using labeled bottles for each participant and its consumption was allowed ad-libitum. Percentage dehydration was calculated as the difference between pre- and post-trial BW relative to pre-trial body mass. For each 5 km split, pacing was calculated as the percentage of speed variation of each split in relation to the mean speed for the 30 km (i.e., positive numbers implies that runners were faster than the mean speed and negative numbers implies that runners were slower than mean speed) [38]. ΔSPEED was calculated as the percentage of speed variation of each split in relation with the MAS (ΔSPEED = MAS—split speed), ΔCMJ was calculated as CMJ height registered at the end of each split minus the baseline height, and ΔCMJ% was defined as percentage of change in CMJ height in relation with baseline CMJ.

### Statistical Analysis

Descriptive statistics were performed using mean, standard deviations and 95% confidence intervals. Normal distribution of the variables was assessed for each data point using the Shapiro-Wilk test and visual inspection of Q-Q plots. For those variables showing a non-normal distribution (e.g, lactate) a natural logarithmic transformation was used, meanwhile those variables showing a non-normal distribution in spite of transformation (i.e., PSE and HR), were assessed using non-parametric techniques (Mann-Whitney U Test for pairwise comparisons and Friedman Test for repeated measures). To assess the effects of time (i.e., stage: 5, 10, 15, 20, 25 and 30 km) an ANOVA for repeated measures was performed. Mauchly’s Sphericity was tested and if sphericity could not be assumed, then the Greenhouse-Geisser correction was used. For repeated measures, a Bonferroni post-hoc comparison was used to locate significant differences. Differences between the 1^st^ and 2^nd^ CMJ in each split were assessed using paired *t* tests. Also, the effect size for each condition was tested by Cohen’s d. Coefficient of variation (CV) was calculated for each variable at all 5 km splits and was averaged to obtain a mean for the sample. Pearson correlations were performed between different variables and such correlations were thereafter assessed for chances of a true effect using qualitative statistics [39] with the following thresholds: *most unlikely*, <0.5%*; very unlikely*, 0.5–5%; *unlikely*, 5–25%; *possibly*, 25–75%; *likely*, 75–95%; *very likely*, 95–99.5%; and *most likely*, >99.5%. All statistics were performed using IBM SPSS Statistics for Windows^®^ (Version 20.0; Armonk, NY). The statistical significance was set at an alpha level of 0.05.

## Results

The trial was completed in 118.5 ± 6.0 min with a mean speed of 3.87 ± 0.49 m·s^−1^ (15.2 ± 0.7 km·h^−1^). Table 2 shows the comparison of the different parameters obtained along the trial. Speed was constant from the start until a decrease was observed after the 4^th^ split (p<0.001; ES = 0.6 to 2.2). The trend for pacing and speed expressed as %MAS was the same as for speed (p<0.001; ES = 0.7 to 1.5; Table 2). Additionally, speed expressed as a %MAS showed an increase at the end of the 2^nd^ split (p<0.001; ES = 0.2). CMJ height increased from baseline (p<0.05, ES = 0.6 to 0.9) and thereafter it was maintained until the end of the trial. Interestingly, CMJ height increased in the 5^th^ split and its value was significantly different from split 4^th^ (p<0.05, ES = 0.2). There were no differences between the 1^st^ and 2^nd^ CMJ assessed within each split. RPE increased at the end of 4^th^ split and reached the highest value at the end of the 6^th^ split. HR increased significantly at the 2^nd^ in comparison with the 1^st^ split (p<0.05, ES = 0.6) and was maintained thereafter while HR expressed as a percentage of the HRmax showed no variation along the trial. Compared to baseline, bLa was higher until the 4^th^ split (p<0.05, ES = 1.7 to 2.6) followed by a decrease at the 6^th^ split, which was lower than the 4^th^ split (p<0.05, ES = 0.7).

**Table 2:** Comparison of the values [Mean ± SD and (95%CI)], and coefficient of variation (CV) for speed, pacing, speed as a % of the maximum aerobic speed (%MAS), countermovement jump height in absolute (CMJ) and relative (ΔCMJ%) terms, lactate (bLa), perceived exertion (RPE), and heart rate (HR), heart rate as a percentage of the maximal (%HRmax) and heart rate as a percentage of the reserve heart rate (%HRR) along the different stages of the trial in half marathon runners.

	Pre	5 km	10 km	15 km	20 km	25 km	30 km	CV
Speed (km·h^−1^)		15.6 ± 0.8 (15.1 to 16.2)	16.0 ± 0.6 (15.6–16.4)	15.7 ± 1.0 (15.0–16.4)	15.3 ± 1.1(14.6–16.1)	14.6 ± 1.2‡§¥ (13.8–15.5)	14.3 ± 0.9†‡ (13.7–14.9)	0.06 ± 0.02
Pacing (%)		2.95 ± 5.64 (-0.84 to 6.74)	5.32 ± 3.26 (3.14 to 7.52)	3.08 ± 3.84 (0.50 to 5.67)	0.61 ± 3.17 (-1.52 to 2.75)	-3.90 ± 3.58‡§¥ (-6.30 to -1.50)	-6.04 ± 5.41†‡ (-9.74 to -2.33)	29.47 ± 26.21
%MAS		77.00 ± 7.00 (72.30 to 81.70)	78.69 ± 5.39† (75.07 to 82.32)	76.96 ± 4.61 (73.86 to 80.07)	75.12 ± 4.21 (72.30 to 77.96)	71.79 ± 4.85‡§¥ (68.53 to 75.06)	70.19 ± 5.79 †‡§ (66.30 to 74.09)	0.06 ± 0.02
CMJ (cm)	23.7 ± 3.3 (21.5 to 25.9)	27.1 ± 3.8@ (24.6 to 29.7)	27.0 ± 5.1@ (23.6 to 30.5)	26.9 ± 5.8@ (23.0 to 30.8)	27.3 ± 6.4@ (23.0 to 31.7)	28.5 ± 6.9@¥ (23.9 to 33.1)	27.2 ± 6.5@ (22.8 to 31.6)	0.09 ± 0.04
ΔCMJ%		15.09 ± 11.29 (7.50 to 22.68)	14.21 ± 12.93 (5.52 to 22.91)	12.88 ± 13.80 (3.61 to 22.16)	14.66 ± 15.84 (4.02 to 25.31)	19.96 ± 20.12¥ (6.45 to 33.49)	14.24 ± 18.24 (1.99 to 26.50)	-3.01 ± 9.01
bLa (mmol·L^−1^)	1.64 ± 0.72 (1.03 to 2.24)	4.53 ± 1.97@ (3.29 to 6.81)	4.59 ± 1.74@ (3.07 to 6.11)	4.76 ± 1.53@ (4.32 to 5.61)	3.54 ± 1.40@ (2.86 to 4.49)	3.16 ± 2.08 (1.73 to 5.52)	2.56 ± 1.43§ (1.53 to 4.19)	0.45 ± 0.14
RPE#		8 ± 2 (7 to 10)	11 ± 2 (9 to 13)	12 ± 2 (11 to 14)	14 ± 2† (12 to 15)	14 ± 3†‡ (12 to 17)	17 ± 2†‡§ (16 to 18)	0.26 ± 0.09
HR (bpm)		160 ± 14 (151 to 169)	168 ± 12† (160 to 177)	168 ± 12 (160 to 177)	168 ± 11 (160 to 176)	166 ± 13 (157 to 174)	165 ± 10 (159 to 173)	0.04 ± 0.02
%HRmax		87 ± 7 (82 to 91)	91 ± 7 (86 to 96)	91 ± 6 (87 to 95)	91 ± 5 (87 to 94)	90 ± 6 (85 to 94)	90 ± 4 (87 to 92)	0.04 ± 0.02
%HRR		80.6 ± 8.7 (74.8 to 86.5)	87.0 ± 9.4 (80.8 to 93.3)	87.0 ± 7.5 (82.0 to 92.0)	86.6 ± 7.2 (81.8 to 91.5)	84.9 ± 9.1 (78.9 to 91.0)	85.0 ± 5.7 (81.2 to 88.8)	0.06 ± 0.04

^#^ Mann-Whitney U Test. @ Significantly different from PRE (p<0.05).

^†^Significantly different from the same value at the 5 km stage (p<0.05).

^‡^Significantly different from the same value at the 10 km stage (p<0.05).

^§^Significantly different from the same value at the 15 km stage (p<0.05).

^¥^Significantly different from the same value at the 20 km stage (p<0.05).

Lactate was analyzed using a natural logarithmic transformation, but the raw data are presented for clarity purposes. Pacing was calculated as the percentage of speed variation in each split in relation to the mean total speed for the 30 km (i.e., positive numbers implies that runners were faster than the mean speed and negative numbers implies that runners were slower than mean speed).

The mean body mass loss during the 30 km trial was 2.5 kg, representing a fluid loss of ~3.5%BM while fluid intake was 0.90 ± 0.3 L (0.3 to 1.6 L) yielding a carbohydrate intake of 53.8 ± 20.3 g (18.0 to 99.0 g).

**Fig 2:**
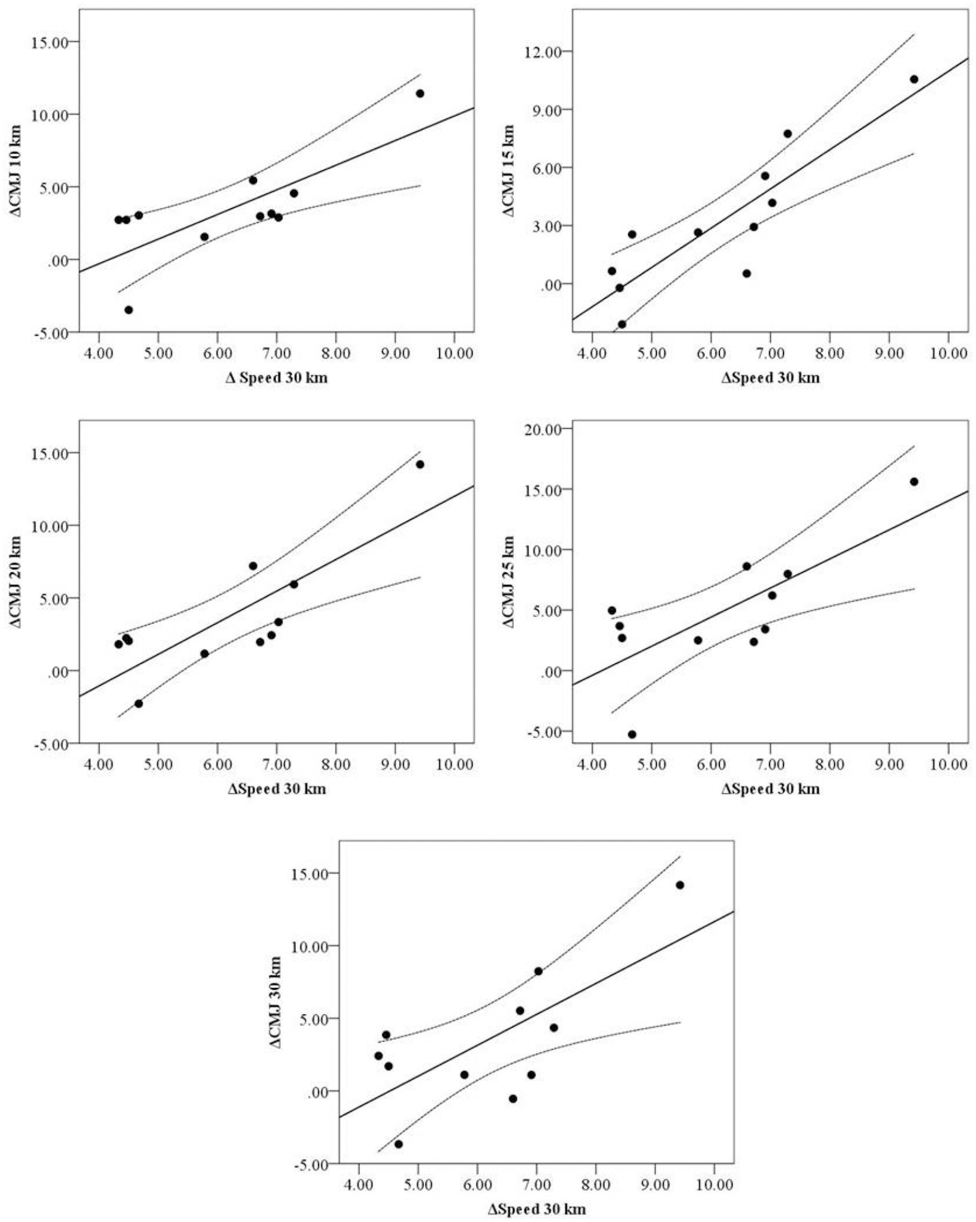
Correlations with 95% confidence limits between ΔCMJ (CMJ height at the end of each split minus CMJ height at baseline) at 10, 15, 20, 25 and 30 km and ΔSPEED 30 km (MAS minus Speed at the 6^th^ split).

**Fig 3:**
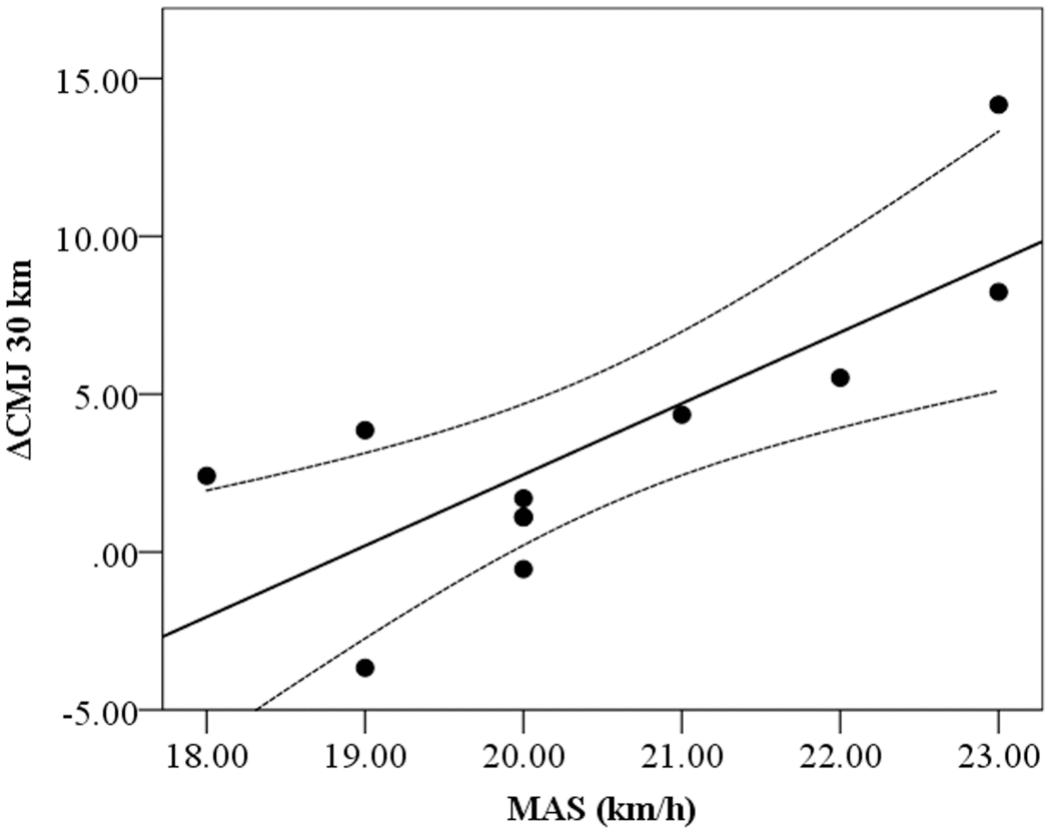
Correlation with 95% confidence limits between ΔCMJ at 30 km and maximal aerobic speed (MAS).

Significant correlations were found between ΔCMJ at 10, 15, 20, 25 and 30 km and ΔSPEED at the 30 km (Fig 2). Qualitative analysis showed that chances of a true effect in these correlations ranged between very likely (98.7%) to most likely (99.9%). Other significant correlations between ΔCMJ and ΔSPEED were: ΔCMJ at 10 km vs ΔSPEED at 20 and 25 km (r = 0.63, p = 0.036 and r = 0.77, p = 0.006, respectively) and ΔCMJ at 15 km vs ΔSPEED at 20 and 25 km (r = 0.65, p = 0.029 and r = 0.77 p = 0.006, respectively). No other significant correlations were found between ΔCMJ and ΔSPEED for the rest of the splits. Also, significant and inverse correlations were found between RPE and speed (Table 3) at different splits and between MAS and ΔCMJ at 30 km (r = 0.77, p<0.05; Fig 3). Additionally, significant correlations were found between MAS and Speed at 15, 20, 25 km (r = 0.67 to 0.73, p<0.05).

**Table 3:** Correlations between rate of perceived exertion (RPE) and speed at different splits along the trial.

	SPEED 5 km	SPEED 10 km	SPEED 15 km	SPEED 20 km	SPEED 25 km	SPEED 30 km
RPE 5 km	-0.140	-0.487	-0.823**	-0.670*	-0.614*	-0.065
RPE 10 km	0.353	-0.563	-0.711*	-0.765**	-0.582	-0.114
RPE 15 km	0.185	-0.780**	-0.695*	-0.787**	-0.574	-0.037
RPE 20 km	0.191	-0.526	-0.620*	-0.667*	-0.572	-0.191
RPE 25 km	0.102	-0.455	-0.398	-0.667*	-0.658*	-0.528
RPE 30 km	0.158	-0.057	0.330	-0.153	-0.201	-0.688*

*Significant at p<0.05.

**Significant at p<0.01.

**Fig 4:**
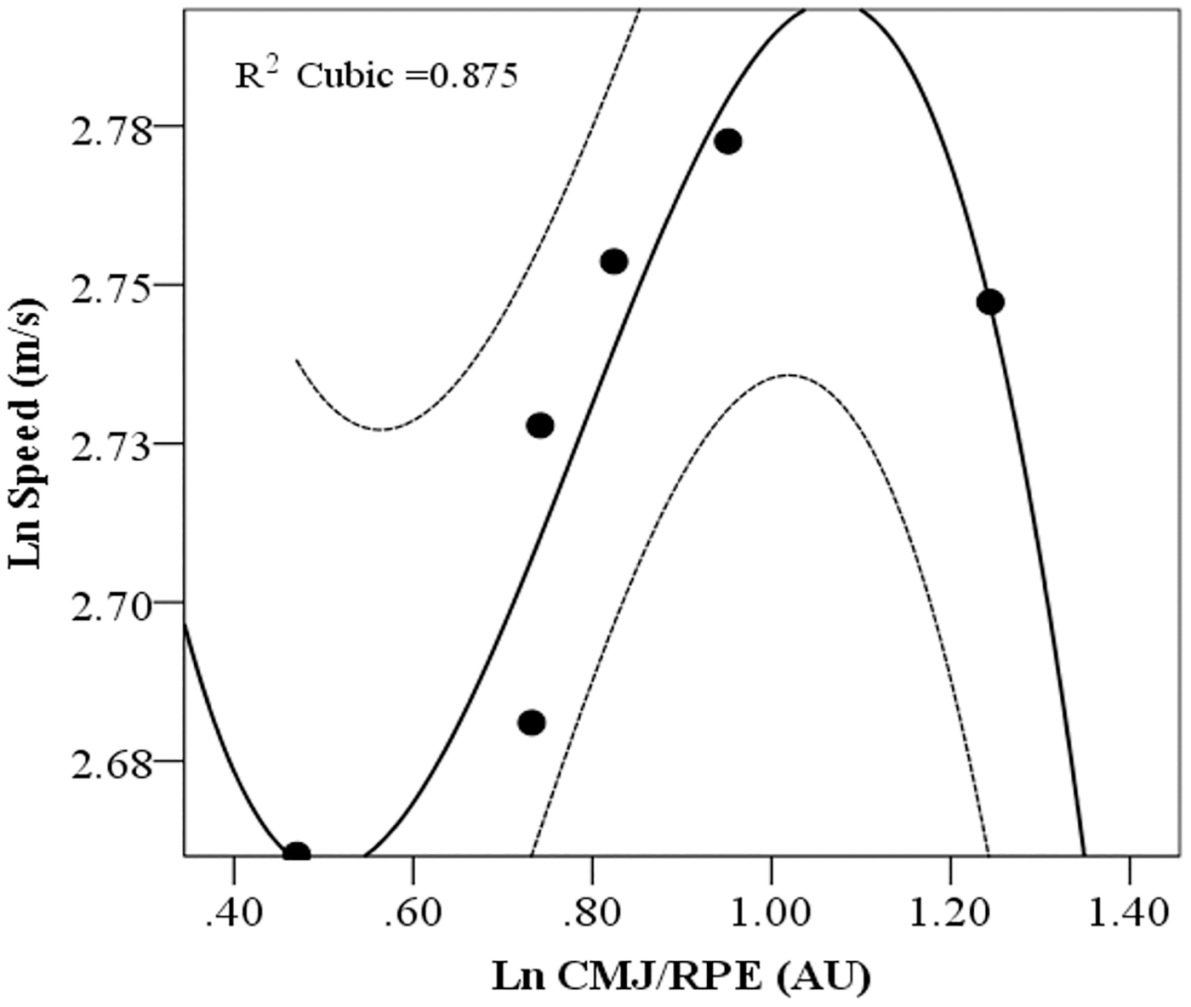
CMJ/RPE index vs. Speed. Values are the natural logarithm of the mean for each split. The data was modeled with a 3^rd^ order polynomial (y = -1.5165x^3^ + 3.5764x^2^–2.4485x + 3.1776; r^2^ = 0.875). The order of splits is reversed due to the logarithmic transformation; therefore the first split is the one on the farthest right.

An index that included both CMJ height and RPE score as CMJ/RPE was calculated and plotted this index vs. the speed along the trial. The natural logarithm of mean values in each stage for CMJ/RPE vs. speed showed a 3^rd^ order fit (Fig 4; y = -1.5165x3 + 3.5764x2–2.4485x + 3.1776; r^2^ = 0.87).

## Discussion

To the best of our knowledge this is the first study describing pacing and concurrent fatigue and potentiation during a self-paced 30 km trial run in endurance athletes. The main findings were: a decrease in speed and thus in pacing from the 4^th^ split until the end of the trial, concomitant with an increase in CMJ from baseline followed by a maintenance of the jump performance; and the relationships between jump potentiation and speed decrements over the running trial. In addition, cardiovascular responses did not change during the trial while the metabolic responses showed a decline (i.e., ~53% in bLa levels) towards the end of the 30 km. Furthermore, RPE scores did not change until the end of the 20 km split. These results of a progressive decline in speed and increase in RPE throughout the trial are in line with previous studies [7, 8]. On the other hand, the increase and maintenance of CMJ height along the trial appears to be related to a potentiation effect as described earlier in endurance athletes [19, 20]

In long distance running, it has been suggested that pacing is regulated by the sensation of fatigue, where RPE acts to evaluate the perception of exertion during exercise [4, 8, 40]. It would appear that athletes adjust their running speed by comparing moment-to-moment the actual with the desirable RPE for a given distance [6, 8] based on an exercise template in the brain that is updated by previous experience and regulates exercise intensity. Our findings support such a contention given that the speed reduction observed in our runners coincided with the increase in RPE (Table 2). Furthermore, there is evidence that pacing is influenced by internal (e.g., glycogen levels) and external (e.g., environmental conditions, previous experience and competitions) cues [41]. In the present study, we observed an increase in bLA during the first splits and a decrease towards the end of the trial (Table 2). In this sense; being that carbohydrate availability appears to be critical in long-term events (> 90 min) [7], it is possible that speed reduction and RPE increases could be related to mechanisms associated with glycogen depletion (not monitored in the present study). It is worth noting that in our study participants had a fluid consumption of 0.90 ± 0.3 L (0.3 to 1.6 L) yielding a carbohydrate intake of 53.8 ± 20.3 g (18.0 to 99.0 g) during the entire trial. Also, there were no correlations between carbohydrate intake and the different measured variables. Recently, Gonzalez et al., [42] showed that carbohydrate ingestion did not prevent muscle glycogen depletion during prolonged cycling (3 h-bouts, 50% of peak power output). In fact, carbohydrate intake in the study of Gonzalez et al., [42] (1.7 g·min^−1^) was larger than the observed in our study (~0.45 g·min^−1^). Thus, it is likely that carbohydrate intake in our study had no substantial effect on muscle glycogen levels. Thus, the classic results from Karlsson and Saltin [43] of different pacing patterns in glycogen loaded vs. not loaded 30 km racers, argues in favor of the decrease in speed being attributable to the depletion of muscle glycogen.

While the previous rationale could partially explain the pacing regulation throughout the trial, it would not explain the increase in CMJ performance observed after the first 10 km and the subsequent maintenance of the jump potentiation (Table 2). In other words, despite the clear evidence of fatigue (decreased speed, elevated RPE), PAP was present throughout the trial. As previously noted, the study of muscle fatigue should address both the perceived effort and decline in force that occurs during sustained activity [10]. In this context, it has been shown that improving the neuromuscular characteristics of endurance athletes would lead to an improvement in performance. In fact, Damasceno et al., [44] observed that improvement in neuromuscular characteristics (i.e., half squat maximum dynamic strength and drop jump height) in recreational long distance runners were accompanied by a faster end-spurt and improvement of performance for the same RPE during a 10 km running time trial. These authors suggested that a possible explanation for the enhanced performance could be a higher motor unit synchronization that might result in strength potentiation and thus delay the onset of signals related to fatigue. In this context, it has been shown that endurance athletes can exhibit concurrent fatigue and potentiation [19, 20]. Moreover, as skeletal muscles can respond to homeostatic disturbances by initiating a facilitatory response to maintain force output [25] it is also possible that neural potentiation from both supraspinal and afferent input could contribute to the maintenance of force production. It can be argued that the threshold to recruit motor units is reduced with neural potentiation and thus fast twitch motor units might be easier to recruit allowing the improvement or maintenance of muscle performance (i.e., jump height and running speed) [25, 45]. Thus, our finding of an increase of CMJ performance could be reflecting a mechanism by which potentiation counteracts fatigue during prolonged exercise. Another appealing candidate for further examination is what has been termed a “peripheral governor” in skeletal muscle [46]. Recently, Froyd et al., [30] assessed potentiation and fatigue during an isokinetic knee extension/flexion time trial and found a significant correlation between indices of fatigue (i.e., post-exercise peak torque) and potentiation (i.e., post-exercise peak torque/pre-exercise peak torque), which would suggest that the same biological mechanisms that cause potentiation (changes in Ca^2+^ sensitivity) may also explain the development and recovery of peripheral fatigue. Thus, based on these previous findings and our results, it could be possible to suggest that pacing may be regulated at both central and peripheral level.

In line with the previous discussion, we found correlations between ΔCMJ at 10, 15, 20, 25 and 30 km and ΔSPEED at 30 km (Fig 2) meaning that those who exhibited the greater improvement in jumping performance (i.e., potentiation) also showed the greatest decrease in speed during the last split. While this could argue against our hypothesis that PAP moderates the effect of fatigue this also could be related to the fact that those running at higher intensities are more likely to exhibit potentiation [29] but in time are more prone to express muscular fatigue. The correlations between MAS and ΔCMJ at 6^th^ split (Fig 3) and between MAS and Speed at 3^rd^, 4^th^ and 5^th^ split (r = 0.67 to 0.73, p<0.05) support this contention. Previously, Vuorimaa et al., [20] found a significant correlation between the enhancement of neuromuscular parameters during fatigue state and MAS in elite long distance runners which is consistent with our finding. More recently, Mettler and Griffin [47] have shown that muscular endurance did not change the pattern of motor unit firing rates but increased endurance time during a fatiguing task. As a partial explanation, the authors [47] suggested that endurance training allowed the central nervous system to sustain similar firing rates of motor units over a longer period of time. In this context, our data shows a clear breaking point after the 4^th^ split (20 km), with a decrease in speed concomitant with an increase in CMJ height and RPE values (Table 2). In this sense, after a 30 km trial, Millet et al., [28] showed that subjects with the greatest knee extensors strength loss also experienced a large activation deficit, which was related with central fatigue. Additionally Millet et al [28] did not find changes in low frequency fatigue after a 30 km running trial and suggested that exercise may have potentiated the contraction torque and hidden the low frequency fatigue. Therefore, taking into account the current and previous findings, we may suggest that the observed increases in jumping performance through the trial may reflect a potentiation effect counterbalancing the effects of fatigue.

We also found an inverse correlation between RPE and speed at different splits (see Table 3). Previously, Crewe et al., [48] reported that the rise in RPE accurately predicted the duration of fixed-intensity exercises and suggested that the brain must be able to forecast the duration of exercise and then set the rate of increase in RPE. This appears to be true for our athletes as some correlations were observed between RPE at 5 km and speeds at 15–25 km, although it has been noticed that pace regulation based on effort perception can result in slower speeds with findings showing that altering perceptions of pace control have an impact on runners’ focus of attention [49]. On one hand these results could be related to the involvement of afferent information [14] as it has been noted that stimulation of III/IV muscle afferents contributes to the prevention of peripheral fatigue during exercise [13]. However, it has also been noticed that afferent feedback does not contribute significantly to perception of effort during exercise and other mechanisms, such as centrally generated forwarding neural signals, may be involved [50]. On the other hand, given that maximal voluntary muscle activation is considered a key indicator of central fatigue, our results of a maintained and increased jumping performance could suggest that PAP is acting to maintain muscle performance. This is in agreement with the results of a previous study [51] showing that endurance-trained athletes can maintain or increase vertical jump performance despite the fatigue induced by an interval training protocol through the improvement of mechanical variables via PAP. Consequently, considering all factors, it is tempting to suggest that in spite of the increase in RPE, the observed increase in CMJ height could be reflecting a peripheral mechanism counteracting the imbalance between fatigue and potentiation and thus a further loss in speed (i.e., greater than the observed decrease). In other words, the neuromuscular system must balance conflicting facilitatory and inhibitory responses in maintaining submaximal forces [25].

Based on the premise that, on one hand maintenance or increased jump performance would be associated with an increase or maintenance of speed and, on the other hand the elevated RPE due to fatigue would be related to a decrease in speed, we calculated an index that included both CMJ height and RPE score as CMJ/RPE and plotted this index vs. the speed along the trial (Fig 4). Interestingly, the mean values in each stage for CMJ/RPE vs. speed showed a 3^rd^ order fit (r^2^ = 0.87), which might imply that balance between fatigue and potentiation could be modulating pacing. Even more interesting is that the model would predict a tendency towards maintenance or reduced CMJ/RPE index, but with a concomitant increase in speed, which could be due to the balance between fatigue and potentiation.

Although this was a multi-stage trial, it is worth mentioning that this model could be considered valid [32] to assess muscle function and pacing during prolonged endurance exercise in a more ecological condition [52]. In activities lasting between 2 min to several hours it has been suggested that competitors typically employ a U-shaped pacing strategy pattern [4, 53] and recently, Hanley [54] showed that half-marathon runners exhibit a reverse-J pacing profile during competitions with some slight differences between men and women. This, in time is associated with the concept of planned pacing strategies that regulate performance [4, 54, 55]. In this sense, although we did not observe such patterns, it is important to note that in our study the athletes were only males and did not run in groups, whereas the selected distance (i.e., 30 km) was not a common competitive distance (e.g., marathon or half marathon). Thus, while we were not able to gather data with a high level of precision within the splits (i.e., GPS), an end-spurt during the terminal segment of the trial (e.g. last km) might be indicative of an increase in PAP as a countermeasure to fatigue with more studies needed. Moreover, Santos-Lozano et al., [38] reported that top runners were those with lower variability in their speed (i.e., CV). In agreement with this, we found a moderate correlation between the CV for speed and total time (r = 0.64, p = 0.035). Therefore it seems that variability of pacing is an issue to consider when analyzing data from prolonged running.

Finally, given that it is known that fluid loss could influence performance during prolonged events [56] in warm environments, we monitored the hydration status by accounting for fluid ingestion and changes in body mass during the trial. The mean body mass loss during the 30 km run was 2.5 kg representing a fluid loss of 3.5%, which is within the acceptable limits of dehydration [56]. Moreover, recently a meta-analysis by Savoie et al. [57] showed that vertical jumping ability is not altered by hypohydration but suggested that a water loss equivalent to ~3% BW may improve performance in BW-dependent tasks. We found that athletes had no significant differences on hypohydration levels and a covariance analysis using percentage of dehydration as a covariate did not show significant results. Thus, it is possible to assume that in our athletes hypohydration had little or no effects on jumping performance.

### Conclusions

In conclusion, we found a reduction in speed and pacing in the final 10 km of a 30 km trial concomitant with the increase in RPE while jumping performance increased from baseline and was maintained thereafter. From our results, it could be suggested that pacing can be regulated by two different mechanisms—one acting as an integrative central center, as reflected in RPE scores, and other acting at a peripheral level whereby the potentiation response increases muscle performance. The 3^rd^ order polynomial fit of the CMJ/RPE index vs. Speed, could be representative of the balance between fatigue and potentiation showing a dual control of the pacing.

## References

[1] Foster C, Snyder AC, Thompson NN, Green MA, Foley M, Schrager M. Effect of pacing strategy on cycle time trial performance. Med Sci Sports Exerc. 1993;25(3):383–8. Epub 1993/03/01. . 8455455

[2] Foster C, De Koning JJ, Hettinga F, Lampen J, La Clair KL, Dodge C, et al Pattern of energy expenditure during simulated competition. Med Sci Sports Exerc. 2003;35(5):826–31. 10.1249/01.MSS.0000065001.17658.68 . 12750593

[3] Tucker R, Noakes TD. The physiological regulation of pacing strategy during exercise: a critical review. Br J Sports Med. 2009;43(6):e1 Epub 2009/02/20. 10.1136/bjsm.2009.057562 bjsm.2009.057562 [pii]. . 19224909

[4] Roelands B, de Koning J, Foster C, Hettinga F, Meeusen R. Neurophysiological determinants of theoretical concepts and mechanisms involved in pacing. Sports Med. 2013;43(5):301–11. Epub 2013/03/05. 10.1007/s40279-013-0030-4 . 23456493

[5] Abbiss CR, Laursen PB. Describing and understanding pacing strategies during athletic competition. Sports Med. 2008;38(3):239–52. . 18278984 10.2165/00007256-200838030-00004

[6] St Clair Gibson A, Lambert EV, Rauch LH, Tucker R, Baden DA, Foster C, et al The role of information processing between the brain and peripheral physiological systems in pacing and perception of effort. Sports Med. 2006;36(8):705–22. . 16869711 10.2165/00007256-200636080-00006

[7] de Koning JJ, Foster C, Bakkum A, Kloppenburg S, Thiel C, Joseph T, et al Regulation of pacing strategy during athletic competition. PLOS ONE. 2011;6(1):e15863 10.1371/journal.pone.0015863 21283744 PMC3024328

[8] Joseph T, Johnson B, Battista RA, Wright G, Dodge C, Porcari JP, et al Perception of fatigue during simulated competition. Med Sci Sports Exerc. 2008;40(2):381–6. 10.1249/mss.0b013e31815a83f6 . 18202562

[9] Abbiss CR, Laursen PB. Models to explain fatigue during prolonged endurance cycling. Sports Med. 2005;35(10):865–98. Epub 2005/09/27. 35104 [pii]. . 16180946 10.2165/00007256-200535100-00004

[10] Barry BK, Enoka RM. The neurobiology of muscle fatigue: 15 years later. Integr Comp Biol. 2007;47(4):465–73. Epub 2007/10/01. 10.1093/icb/icm047 . 21672855

[11] Renfree A, Martin L, Micklewright D, St Clair Gibson A. Application of decision-making theory to the regulation of muscular work rate during self-paced competitive endurance activity. Sports Med. 2014;44(2):147–58. 10.1007/s40279-013-0107-0 . 24113898

[12] Baron B, Moullan F, Deruelle F, Noakes TD. The role of emotions on pacing strategies and performance in middle and long duration sport events. Br J Sports Med. 2011;45(6):511–7. 10.1136/bjsm.2009.059964 . 19553226

[13] Amann M, Sidhu SK, Weavil JC, Mangum TS, Venturelli M. Autonomic responses to exercise: group III/IV muscle afferents and fatigue. Auton Neurosci. 2015;188:19–23. 10.1016/j.autneu.2014.10.018 25458423 PMC4336599

[14] Amann M. Significance of Group III and IV muscle afferents for the endurance exercising human. Clin Exp Pharmacol Physiol. 2012;39(9):831–5. 10.1111/j.1440-1681.2012.05681.x 22300329 PMC3351566

[15] Millet GY. Can neuromuscular fatigue explain running strategies and performance in ultra-marathons?: the flush model. Sports Med. 2011;41(6):489–506. 10.2165/11588760-000000000-00000 . 21615190

[16] Paavolainen L, Hakkinen K, Hamalainen I, Nummela A, Rusko H. Explosive-strength training improves 5-km running time by improving running economy and muscle power. J Appl Physiol (1985). 1999;86(5):1527–33. . 10233114 10.1152/jappl.1999.86.5.1527

[17] Berryman N, Maurel D, Bosquet L. Effect of plyometric vs. dynamic weight training on the energy cost of running. J Strength Cond Res. 2010;24(7):1818–25. 10.1519/JSC.0b013e3181def1f5 . 20543734

[18] Beattie K, Kenny IC, Lyons M, Carson BP. The effect of strength training on performance in endurance athletes. Sports Med. 2014;44(6):845–65. 10.1007/s40279-014-0157-y . 24532151

[19] Boullosa DA, Tuimil JL, Alegre LM, Iglesias E, Lusquinos F. Concurrent fatigue and potentiation in endurance athletes. Int J Sports Physiol Perform. 2011;6(1):82–93. . 21487152 10.1123/ijspp.6.1.82

[20] Vuorimaa T, Virlander R, Kurkilahti P, Vasankari T, Hakkinen K. Acute changes in muscle activation and leg extension performance after different running exercises in elite long distance runners. Eur J Appl Physiol. 2006;96(3):282–91. 10.1007/s00421-005-0054-z . 16283372

[21] Tillin NA, Bishop D. Factors modulating post-activation potentiation and its effect on performance of subsequent explosive activities. Sports Med. 2009;39(2):147–66. 10.2165/00007256-200939020-00004 . 19203135

[22] Rassier DE, Macintosh BR. Coexistence of potentiation and fatigue in skeletal muscle. Braz J Med Biol Res. 2000;33(5):499–508. . 10775880 10.1590/s0100-879x2000000500003

[23] MacIntosh BR. Role of calcium sensitivity modulation in skeletal muscle performance. News Physiol Sci. 2003;18:222–5. . 14614153 10.1152/nips.01456.2003

[24] Sale D. Postactivation potentiation: role in performance. Br J Sports Med. 2004;38(4):386–7. 10.1136/bjsm.2002.003392 15273166 PMC1724858

[25] Behm DG. Force maintenance with submaximal fatiguing contractions. Can J Appl Physiol. 2004;29(3):274–90. . 15199227 10.1139/h04-019

[26] Boullosa DA, Tuimil JL. Postactivation potentiation in distance runners after two different field running protocols. J Strength Cond Res. 2009;23(5):1560–5. 10.1519/JSC.0b013e3181a3ce61 . 19620906

[27] Garcia-Pinillos F, Soto-Hermoso VM, Latorre-Roman PA. Acute effects of extended interval training on countermovement jump and handgrip strength performance in endurance athletes: postactivation potentiation. J Strength Cond Res. 2015;29(1):11–21. 10.1519/JSC.0000000000000591 . 25532430

[28] Millet GY, Martin V, Lattier G, Ballay Y. Mechanisms contributing to knee extensor strength loss after prolonged running exercise. J Appl Physiol (1985). 2003;94(1):193–8. 10.1152/japplphysiol.00600.2002 . 12391039

[29] Mettler JA, Griffin L. Postactivation potentiation and muscular endurance training. Muscle Nerve. 2012;45(3):416–25. 10.1002/mus.22313 . 22334177

[30] Froyd C, Beltrami FG, Jensen J, Millet GY, Noakes TD. Potentiation and electrical stimulus frequency during self-paced exercise and recovery. J Hum Kinet. 2014;42:91–101. 10.2478/hukin-2014-0064 25414743 PMC4234774

[31] Morana C, Perrey S. Time course of postactivation potentiation during intermittent submaximal fatiguing contractions in endurance- and power-trained athletes. J Strength Cond Res. 2009;23(5):1456–64. 10.1519/JSC.0b013e3181a518f1 . 19620919

[32] McIntyre JP, Mawston GA, Cairns SP. Changes of whole-body power, muscle function, and jump performance with prolonged cycling to exhaustion. Int J Sports Physiol Perform. 2012;7(4):332–9. . 22645195 10.1123/ijspp.7.4.332

[33] Borg GA. Psychophysical bases of perceived exertion. Med Sci Sports Exerc. 1982;14(5):377–81. . 7154893

[34] Zouhal H, Ben Abderrahman A, Prioux J, Knechtle B, Bouguerra L, Kebsi W, et al Drafting's improvement of 3000-m running performance in elite athletes: is it a placebo effect? Int J Sports Physiol Perform. 2015;10(2):147–52. 10.1123/ijspp.2013-0498 . 24912074

[35] Leger L, Boucher R. An indirect continuous running multistage field test: the Universite de Montreal track test. Can J Appl Sport Sci. 1980;5(2):77–84. . 7389053

[36] de Blas X, Padullés JM, López del Amo JL, Guerra-Balic M. Creation and validation of Chronojump-Boscosystem: A free tool to measure vertical jumps. Int J Sports Sci. 2012;8:334–56.

[37] Pagaduan JC, De Blas X. Reliability of countermovement jump performance on chronojump-boscosystem in male and female athletes. Sports SPA. 2004;10(2):5–8.

[38] Santos-Lozano A, Collado PS, Foster C, Lucia A, Garatachea N. Influence of sex and level on marathon pacing strategy. Insights from the New York City race. Int J Sports Med. 2014;35(11):933–8. 10.1055/s-0034-1367048 . 24886929

[39] Hopkins WG, Marshall SW, Batterham AM, Hanin J. Progressive statistics for studies in sports medicine and exercise science. Med Sci Sports Exerc. 2009;41(1):3–13. 10.1249/MSS.0b013e31818cb278 . 19092709

[40] Hampson DB, Clair Gibson A St, Lambert MI, Noakes TD. The influence of sensory cues on the perception of exertion during exercise and central regulation of exercise performance. Sports Med. 2001;31(13):935–52. . 11708402 10.2165/00007256-200131130-00004

[41] Jones HS, Williams EL, Bridge CA, Marchant D, Midgley AW, Micklewright D, et al Physiological and psychological effects of deception on pacing strategy and performance: a review. Sports Med. 2013;43(12):1243–57. 10.1007/s40279-013-0094-1 . 24002790

[42] Gonzalez JT, Fuchs CJ, Smith FE, Thelwall PE, Taylor R, Stevenson EJ, et al Ingestion of glucose or sucrose prevents liver but not muscle glycogen depletion during prolonged endurance-type exercise in trained cyclists. Am J Physiol Endocrinol Metab. 2015;309(12):E1032–9. 10.1152/ajpendo.00376.2015 . 26487008

[43] Karlsson J, Saltin B. Diet, muscle glycogen, and endurance performance. J Appl Physiol. 1971;31(2):203–6. . 5558241 10.1152/jappl.1971.31.2.203

[44] Damasceno MV, Lima-Silva AE, Pasqua LA, Tricoli V, Duarte M, Bishop DJ, et al Effects of resistance training on neuromuscular characteristics and pacing during 10-km running time trial. Eur J Appl Physiol. 2015;115(7):1513–22. 10.1007/s00421-015-3130-z . 25697149

[45] Behm DG, Button DC, Barbour G, Butt JC, Young WB. Conflicting effects of fatigue and potentiation on voluntary force. J Strength Cond Res. 2004;18(2):365–72. 10.1519/R-12982.1 . 15141999

[46] MacIntosh BR, Shahi MR. A peripheral governor regulates muscle contraction. Appl Physiol Nutr Metab. 2011;36(1):1–11. 10.1139/H10-073 . 21326373

[47] Mettler JA, Griffin L. Muscular endurance training and motor unit firing patterns during fatigue. Exp Brain Res. 2015 10.1007/s00221-015-4455-x . 26449966

[48] Crewe H, Tucker R, Noakes TD. The rate of increase in rating of perceived exertion predicts the duration of exercise to fatigue at a fixed power output in different environmental conditions. Eur J Appl Physiol. 2008;103(5):569–77. 10.1007/s00421-008-0741-7 . 18461352

[49] Brick NE, Campbell MJ, Metcalfe RS, Mair JL, MacIntyre TE. Altering Pace Control and Pace Regulation: Attentional Focus Effects during Running. Med Sci Sports Exerc. 2015 10.1249/MSS.0000000000000843 . 26673128

[50] Marcora S. Perception of effort during exercise is independent of afferent feedback from skeletal muscles, heart, and lungs. J Appl Physiol (1985). 2009;106(6):2060–2. 10.1152/japplphysiol.90378.2008 . 18483166

[51] Latorre-Román PÁ, García-Pinillos F, Martínez-López EJ, Soto-Hermoso VM. Concurrent fatigue and postactivation potentiation during extended interval training in long-distance runners. Motriz: Revista de Educação Física. 2014;20:423–30. 10.1590/S1980-65742014000400009

[52] Boullosa DA, Nakamura FY. The evolutionary significance of fatigue. Front Physiol. 2013;4:309 10.3389/fphys.2013.00309 24198788 PMC3814088

[53] Tucker R, Marle T, Lambert EV, Noakes TD. The rate of heat storage mediates an anticipatory reduction in exercise intensity during cycling at a fixed rating of perceived exertion. J Physiol. 2006;574(Pt 3):905–15. 10.1113/jphysiol.2005.101733 16497719 PMC1817748

[54] Hanley B. Pacing profiles and pack running at the IAAF World Half Marathon Championships. J Sports Sci. 2015;33(11):1189–95. 10.1080/02640414.2014.988742 . 25483017

[55] Hanley B. Pacing, packing and sex-based differences in Olympic and IAAF World Championship marathons. J Sports Sci. 2016:1–7. 10.1080/02640414.2015.1132841 . 26736042

[56] Noakes TD. Drinking guidelines for exercise: what evidence is there that athletes should drink "as much as tolerable", "to replace the weight lost during exercise" or "ad libitum"? J Sports Sci. 2007;25(7):781–96. 10.1080/02640410600875036 . 17454546

[57] Savoie FA, Kenefick RW, Ely BR, Cheuvront SN, Goulet ED. Effect of Hypohydration on Muscle Endurance, Strength, Anaerobic Power and Capacity and Vertical Jumping Ability: A Meta-Analysis. Sports Med. 2015;45(8):1207–27. 10.1007/s40279-015-0349-0 . 26178327

